# A Combination of Conformation-Specific RAF Inhibitors Overcome Drug Resistance Brought about by RAF Overexpression

**DOI:** 10.3390/biom13081212

**Published:** 2023-08-02

**Authors:** Hiroaki Imoto, Nora Rauch, Ashish J. Neve, Fahimeh Khorsand, Martina Kreileder, Leonidas G. Alexopoulos, Jens Rauch, Mariko Okada, Boris N. Kholodenko, Oleksii S. Rukhlenko

**Affiliations:** 1 Systems Biology Ireland, School of Medicine, University College Dublin, D04 V1W8 Dublin, Ireland; 2 Protavio Ltd., Demokritos Science Park, 153 43 Athens, Greece; 3 Department of Mechanical Engineering, National Technical University of Athens, 106 82 Athens, Greece; 4 School of Biomolecular and Biomedical Science, University College Dublin, D04 V1W8 Dublin, Ireland; 5 Institute for Protein Research, Osaka University, Osaka 565-0871, Japan; 6 Premium Research Institute for Human Metaverse Medicine (WPI-PRIMe), Osaka University, Osaka 565-0871, Japan; 7 Conway Institute of Biomolecular and Biomedical Research, University College Dublin, D04 V1W8 Dublin, Ireland; 8 Department of Pharmacology, Yale University School of Medicine, New Haven, CT 06520, USA

**Keywords:** MAP Kinases, RAF dimerization, RAF inhibitor resistance, structure-based mechanistic modeling, RAF isoforms, ARAF knockout

## Abstract

Cancer cells often adapt to targeted therapies, yet the molecular mechanisms underlying adaptive resistance remain only partially understood. Here, we explore a mechanism of RAS/RAF/MEK/ERK (MAPK) pathway reactivation through the upregulation of RAF isoform (RAFs) abundance. Using computational modeling and in vitro experiments, we show that the upregulation of RAFs changes the concentration range of paradoxical pathway activation upon treatment with conformation-specific RAF inhibitors. Additionally, our data indicate that the signaling output upon loss or downregulation of one RAF isoform can be compensated by overexpression of other RAF isoforms. We furthermore demonstrate that, while single RAF inhibitors cannot efficiently inhibit ERK reactivation caused by RAF overexpression, a combination of two structurally distinct RAF inhibitors synergizes to robustly suppress pathway reactivation.

## 1. Introduction

Pathway reactivation is a key mechanism of both innate and acquired resistance to targeted therapy. Understanding the underlying mechanisms of pathway reactivation is therefore critical for rationally designing effective drugs and treatments in the clinic to overcome drug resistance.

The RAS/RAF/MEK/ERK (MAPK) pathway plays a central role in cellular proliferation, and oncogenic mutations in this pathway are frequently observed. *RAS* (*HRAS, KRAS*, and *NRAS*) is the most frequently mutated gene in human cancers, particularly in positions G12 and Q61, and, consequently, the inhibition of oncogenic signaling mediated by RAS has been a key challenge in the field of cancer biology. Yet, despite more than three decades of painstaking research to develop effective therapeutics against RAS-driven oncogenesis, the inhibition of mutated RAS has been elusive, due to its high affinity for GTP and lack of accessible binding pockets [1]. RAS has only recently become druggable, thanks to the development of covalent inhibitors against the KRAS^G12C^ mutant [2]. However, *KRAS^G12C^* is mainly found in non–small-cell lung cancers (NSCLCs) [3], but rarely in other types of cancers. Therefore, the investigation of new therapeutics that target downstream of RAS (RAF and MEK) has been a hot topic and is still important.

Protein kinases of the RAF family, including ARAF, BRAF, and CRAF, are key signaling molecules downstream of RAS in the RAS/RAF/MEK/ERK pathway [4,5,6]. RAF proteins also contain a high number of oncogenic mutations that bring about constitutive activation of this pathway, with BRAF^V600E^ being the most common [7,8].

In contrast to RAS, oncogenic RAF can successfully be targeted by small-molecule inhibitors. Vemurafenib was identified as a selective inhibitor of BRAF^V600E^ more than a decade ago, and has been proven to work effectively in BRAF mutated melanoma, although resistance to Vemurafenib develops rapidly [9,10,11]. In a RAS mutant context, however, all RAF inhibitors were shown to paradoxically amplify RAS-mediated signaling instead of inhibiting it [6,12,13].

Cancer cells use multiple mechanisms of adaptation to small molecule inhibitors, resulting in drug resistance. For instance, dimerization of RAF molecules facilitated by targeted inhibitors is a well-known mechanism of resistance and hyperactivation of the RAS/RAF/MEK/ERK pathway [6,12,13,14]. The relief of negative feedback is another mechanism for pathway reactivation [15,16]. However, even if negative feedback is present, but a kinase does not dimerize, a complete reactivation of the pathway is impossible [17]. Moreover, even if a kinase dimerizes but inhibitors do not facilitate dimerization, multiple negative and positive feedback loops in a pathway cannot lead to complete, steady state revival of the output activity [17].

In this study, we explore yet another mechanism of pathway reactivation where the cell’s exposure to an inhibitor results in the increase in the abundance of the targeted kinase that can dimerize or oligomerize. Gene amplification and a concomitant increase in BRAF^V600E^ abundance upon RAF inhibitor treatment is often observed in the clinic [18]. Although this mechanism is known to cause resistance, it has never been studied in detail.

Using structure-based modeling and in vitro experiments, we aim to understand how the upregulation of RAF isoforms can change the concentration range of paradoxical activation of the MAPK pathway upon treatment with RAF inhibitors, and how it can result in complete reactivation, or even over-shooting of the ERK signal. We also aim to understand how this resistance mechanism can be overcome with single drugs or their combinations.

## 2. Materials and Methods

### 2.1. Cell Culture

The MEL-JUSO (NRAS^Q61L/WT^, HRAS^G13D/G13D^) cell line was purchased from DSMZ (#ACC 74). The OCI-AML-3 (NRAS^Q61l/Q61L^) cell line was a gift from Prof. Ken Mills, Queen’s University Belfast. CaCo-2tet/HA-BRAF^WT^ cells were kindly provided by Prof. Tilman Brummer, University of Freiburg [19,20]. *ARAF^−/−^* single cell MEL-JUSO clones were generated using the GeneArt^®^ CRISPR Nuclease Plasmid Vector system (Thermo Fisher Scientific, Waltham, MA, USA, #A21174), according to the manufacturer’s instructions. *ARAF* specific gRNA sequences were designed using the GeneArt CRISPR design tool Thermo Fisher Scientific, Waltham, MA, USA). MEL-JUSO and OCI-AML-3 cells were grown in RPMI 1640 supplemented with 2 mM L-glutamine and 10% (*v*/*v*) fetal bovine serum (all all Gibco™, Thermo Fisher Scientific, Waltham, MA, USA) in a humidified atmosphere of 5% CO_2_ at 37 °C. CaCo-2tet/HA-BRAF^WT^ cells were grown in DMEM supplemented with 2 mM L-glutamine and 10% (*v*/*v*) fetal bovine serum (all Gibco™, Thermo Fisher Scientific, Waltham, MA, USA), plus 5 µg/mL puromycin (Sigma-Aldrich, Burlington, MA, USA) and 5 µg/mL blasticdin (all Gibco™, Thermo Fisher Scientific, Waltham, MA, USA). Overexpression of BRAF^WT^ was induced by addition of 2 µg/mL Doxycycline (Sigma-Aldrich, Burlington, MA, USA). For treatments, MEL-JUSO and CaCo-2 cells were seeded in 6-well plates at a density of 3 × 10^5^ cells per well. OCI-AML-3 were seeded in 12-well plates at a concentration of 5 × 10^5^/mL. After reaching sufficient confluency (MEL-JUSO, CaCo-2) respectively cell number (OCI-AML-3), the cells were treated as indicated.

### 2.2. RAF Inhibitors

TAK-632 (#S7291), Vemurafenib (PLX4032, #1267), and Encorafenib (LGX818, #S7108) were purchased from Selleck Chemicals LLC, Houston, TX, USA. Sorafenib tosylate (Axon 1397) was from Axon Medchem, Groningen, The Netherlands, and SB-590885 (2650/10) was purchased from R&D Systems, Minneapolis, MN, USA. Inhibitors were dissolved in DMSO. Stocks were kept at −80 °C and further diluted in DMSO as required.

### 2.3. Western Blot

Total lysates for western blotting were prepared on ice using cold 10 mM Tris-HCl pH 7.5, 150 mM NaCl, and 0.5% (*v*/*v*) NP-40 (Calbiochem, San Diego, CA, USA), complemented with cOmplete™ mini protease inhibitor (#11836170001) and PhosSTOP phosphatase inhibitor (#04906837001) cocktails (both from Roche, Rotkreuz, Switzerland). Following centrifugation at 10,000× *g* at 4 °C for 10 min to remove cell debris, lysates were adjusted to equal protein concentrations using a Pierce™ BCA Protein Assay Kit (Thermo Fisher Scientific, Waltham, MA, USA #23225). Lysates were then resolved by SDS PAGE (10% PAA) in the Mini Protean Tetra system (Bio-Rad, Hercules, CA, USA), and proteins were transferred on a polyvinylidene difluoride membrane (PVDF, MilliporeSigma, Burlington, CA, USA). Protein visualization was performed by the iBright™ CL750 Imaging System (Invitrogen™, Thermo Fisher Scientific, Waltham, MA, USA), using horseradish peroxidase-conjugated secondary antibodies (#7074 resp. #7076, from Cell Signaling Technologies, Danvers, MA, USA), and the enhanced chemiluminescence system (GE Healthcare, Piscataway, NJ, USA) for the following antibodies: ARAF (D2P9P) (#75804); GAPDH (14C10) (#2118), both from Cell Signaling Technologies, Danvers, MA, USA; BRAF (F-7) (# sc-5284); CRAF/RAF1 (C-12) (# sc-133), both from Santa Cruz Biotechnology, Inc., Dallas, TX, USA, 75220; and polyclonal rabbit anti-human mitogen-activated protein (MAP) kinase [extra-cellular signal-regulated kinase (ERK) 1 & 2] antibody (#M5670), monoclonal mouse anti-human MAP kinase, activated (diphosphorylated ERK-1 & 2) antibody (#8159), both from Sigma-Aldrich, Burlington, MA, USA. Blots were quantified using ImageJ/Fiji (version 1.5.4f) [21].

### 2.4. Luminex ELISA

5 × 10^5^ MEL-JUSO cells (*ARAF^+^*^/*+*^ or *ARAF^−/−^*) were seeded in 6-well plates. After 24 h, cells were treated with Vemurafenib, Sorafenib tosylate or DMSO for 24 h. Cells were harvested in MILLIPLEX^®^MAP lysis buffer (1X) supplemented with cOmplete^TM^ mini protease inhibitor (Roche, Rotkreuz, Switzerland #11836170001) according to the manufacturer’s protocol (Cell Signalling Buffer and Detection Kit, Millipore Sigma, Burlington, MA, USA #48-602MAG). Total protein concentrations were adjusted to 0.3 µg/µL using a Pierce™ BCA Protein Assay Kit (Thermo Fisher Scientific, Waltham, MA, USA #23225). MILLIPLEX ELISA assays to assess ERK1/2 phosphorylation (Thr185/Tyr187) and total ERK1/2 levels were performed on a MAGPIX system (Luminex xMAP-technology) using a Phospho/Total ERK 2-Plex Magnetic Bead Kit (MILLIPLEX^®^MAP #48-619MAG), according to the manufacturer’s instructions. Mean Fluorescence Intensity (MFI) measurements were analyzed.

### 2.5. MSD Multi-Spot Assay ELISA System

5 × 10^5^ MEL-JUSO cells were seeded in 6-well plates. After 24hrs, cells were treated with SB-590885 or Sorafenib tosylate, including the DMSO controls, for 1, 2, 6 and 24 h. ERK activation was assessed by ELISA using the MESOSCALE MSD kit (MESO SCALE DIAGNOSTICS, LLC, Rockville, MD, USA #K15107D), according to the manufacturer’s instructions. Briefly, following the addition of complete MSD lysis buffer and scraping the cells from the surface of the dish, the cellular debris was removed from the lysate by centrifugation at 10,000× *g* at 4 °C for 10 min. Protein concentration was determined using the Pierce™ BCA Protein Assay Kit (Thermo Fisher Scientific, Waltham, MA, USA), according to the manufacturer’s instructions. Lysates were adjusted to 0.1 µg/µL protein concentrations and relative ERK activation assessed according to the manufacturer’s instructions using the MSD Sector Imager 2400 (model 1250).

### 2.6. xMAP Assays

5 × 10^5^ MEL-JUSO cells were seeded in 6-well plates. After 24 h, cells were treated with SB-590885 or Sorafenib tosylate, including the DMSO controls, for 24 h. Following the addition of complete Luminex lysis buffer and scraping the cells from the surface of the dish, the cellular debris was removed from the lysate by centrifugation at 10,000× *g* at 4 °C for 10 min. The pellet was discarded, and the protein concentration of lysates was adjusted to 0.3 µg/µL, using the BCA assay kit. The xMAP assays were performed on a Luminex-3D platform (Luminex, Austin, TX, USA), using commercially available phosphoprotein antibody-coupled beads (ProtATonce, Athens, Greece). A custom multiplex phosphoprotein assay was used to determine the levels of test phosphoproteins in cell lysates: extracellular signal-regulated kinase-1 (ERK1) with phosphorylation site T202/Y204. Additionally, for loading control, the levels of glyceraldehyde 3-phosphate dehydrogenase (GAPDH) protein were analyzed in a separate setting. Custom antibody-coupled beads were technically validated as described before [22].

### 2.7. Cell Viability Assays

Cell proliferation of *ARAF^+/+^* and *ARAF^−/−^* MEL-JUSO cells was analyzed by CellTiter 96 Aqueous One Solution Cell Proliferation Assay (MTS; Promega), according to the manufacturer’s instructions. For this, cells were plated in 96-well tissue culture plates (in 200 µL of medium) and treated with RAFi. Cell proliferation/viability of inhibitor- and control-treated cells assayed after 72 h. The results represent the mean ± SD of triplicate biological samples, expressed as a percentage of control.

### 2.8. A Core Mathematical Model of Enzyme-Inhibitor Interaction

**Figure 1:**
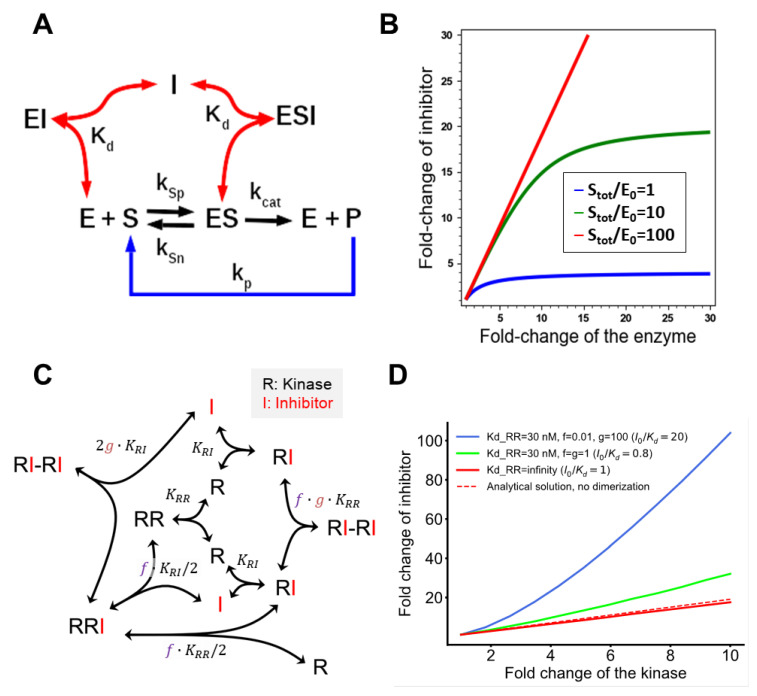
Increase in inhibitor doses required for constant inhibition of a kinase following its overexpression: insight from simple kinetic models. (**A**) Mass action scheme of phosphorylation of the substrate S by kinase E, which is inhibited by non-competitive inhibitor I. (**B**) Fold-changes in the drug dose (e.g., IC50) required to maintain the same inhibition level (e.g., 50%) are presented versus fold-changes in the abundance of the primary target for different enzyme/substrate ratios (see Section 2, Materials and Methods). (**C**) Schematic illustration of the kinase dimer model derived from [14]. (**D**) Increase in the IC50 drug dose following the increase in the abundance for a kinase that can dimerize in cases where the inhibitor induces dimerization (blue) or does not induce dimerizaton (lime green), compared to a kinase that does not dimerize (red). Calculated using the model presented in [14]. The dashed line (analytical solution, no dimerization) denotes the linear relationship between the drug dose and the abundance of a kinase derived from Equation (11).

Overexpression of a targeted enzyme requires an increase in the drug dose. To illustrate a simple case, when the enzyme does not dimerize, a core model calculates the increase in IC50 drug dose (or a dose needed to achieve a desired inhibition level) for the given increase in enzyme abundance. In the mass action scheme (Figure 1A), a kinase (E) phosphorylates its protein substrate (S), yielding the product (P), which, in turn, is dephosphorylated with the rate constant $k_{p}$. An inhibitor (I) binds to both the free kinase and the substrate-bound kinase (ES) with the dissociation constant $K_{d}$ (non-competitive inhibition). The rate constants $k_{Sp}$ and $k_{Sn}$ are the elementary association and dissociation constants, and $K_{S}=k_{Sn}/k_{Sp}$ is the equilibrium dissociation constant. The rate constant $k_{cat}$ describes the catalytic step.

Given that the dose $I_{0}={IC50}_{0}$ leads to a 50% inhibition of the kinase when its abundance is $E_{0}^{tot}$, the following equations describe the moiety conservation laws at the system’s steady state:

(1) $$E_{0}\left(\frac{I_{0}}{K_{d}}+1\right)\left(\frac{S_{0}}{K_{S}}+1\right)=E_{0}^{tot}\;S_{0}\left(1+\frac{E_{0}}{K_{S}}\left(1+\frac{k_{cat}}{k_{p}}+\frac{I_{0}}{K_{d}}\right)\right)=S^{tot}$$

Solving these equations, we obtain concentrations of the free kinase $E_{0}$ and its substrate $S_{0}$ as functions $f_{E0}$ and $f_{S0}$ of the total abundances, $E_{0}^{tot}$, $S^{tot}$, $I_{0}={IC50}_{0}$, and parameters:

(2) $$E_{0}=f_{E0}\left(\frac{I_{0}}{K_{d}},K_{S},\frac{k_{cat}}{k_{p}},E_{0}^{tot},S^{tot}\right)\;S_{0}=f_{S0}\left(\frac{I_{0}}{K_{d}},K_{S},\frac{k_{cat}}{k_{p}},E_{0}^{tot},S^{tot}\right)$$

When the kinase (E) is overexpressed L-fold, an F-fold concentration increase in an inhibitor is needed to achieve the same level of inhibition. After a kinase is overexpressed, the moiety conservation laws for the overexpressed kinase and its substrate read as follows:

(3) $$E\left(\frac{FI_{0}}{K_{d}}+1\right)\left(\frac{S}{K_{S}}+1\right)=LE_{0}^{tot}\;S\left(1+\frac{E}{K_{S}}\left(1+\frac{k_{cat}}{k_{p}}+\frac{FI_{0}}{K_{d}}\right)\right)=S^{tot}$$

Solving these equations, we obtain the concentrations of the free kinase E and the substrate S as functions $f_{E}$ and $f_{S}$ of the new parameters, $LE_{0}^{tot}$ and $FI_{0}$:

(4) $$E=f_{E}\left(\frac{FI_{0}}{K_{d}},K_{S},\frac{k_{cat}}{k_{p}},LE_{0}^{tot},S^{tot}\right)\;S=f_{S}\left(\frac{FI_{0}}{K_{d}},K_{S},\frac{k_{cat}}{k_{p}},LE_{0}^{tot},S^{tot}\right)$$

The F-fold increase in inhibitor concentration must yield the same 50% level of kinase inhibition, i.e., the same level of substrate phosphorylation, as follows:

(5) $$k_{cat}\cdot E\cdot S=k_{cat}\cdot E_{0}\cdot S_{0}$$

Substituting Equations (3) and (4) into Equation (5), we finally obtain:

(6) $$f_{E}\left(\frac{FI_{0}}{K_{d}},K_{S},\frac{k_{cat}}{k_{p}},LE_{0}^{tot},S^{tot}\right)\cdot f_{S}\left(\frac{FI_{0}}{K_{d}},K_{S},\frac{k_{cat}}{k_{p}},LE_{0}^{tot},S^{tot}\right)=\;f_{E0}\left(\frac{I_{0}}{K_{d}},K_{S},\frac{k_{cat}}{k_{p}},E_{0}^{tot},S^{tot}\right)\cdot f_{S0}\left(\frac{I_{0}}{K_{d}},K_{S},\frac{k_{cat}}{k_{p}},E_{0}^{tot},S^{tot}\right)$$

Equation (6) cannot be solved analytically. We utilized computer algebra system *Sage* [23,24] for implicit plotting of the dependence of F on L.

However, we can explicitly solve Equation (6) for specific cases. First, we assume that the kinase abundance is smaller than the substrate abundance both before, $E_{0}^{tot}\ll S^{tot}$, and, after its overexpression, $LE_{0}^{tot}\ll S^{tot}$. Then, $S\approx S^{tot}$, and the moiety conservation equations for the kinase before and after overexpression read as follows:

(7) $$E_{0}\left(\frac{I_{0}}{K_{d}}+1\right)\left(\frac{S^{tot}}{K_{S}}+1\right)=E_{0}^{tot}\;\to \;\;E_{0}=\frac{E_{0}^{tot}}{\left(\frac{I_{0}}{K_{d}}+1\right)\left(\frac{S^{tot}}{K_{S}}+1\right)}\;E_{0}\left(\frac{FI_{0}}{K_{d}}+1\right)\left(\frac{S^{tot}}{K_{S}}+1\right)=LE_{0}^{tot}\;\;\to \;\;E=\frac{LE_{0}^{tot}}{\left(\frac{FI_{0}}{K_{d}}+1\right)\left(\frac{S^{tot}}{K_{S}}+1\right)}$$

The equality of the phosphorylation rates Equation (6) read as follows:

(8) $$k_{cat}\cdot \frac{LE_{0}^{tot}}{\left(\frac{FI_{0}}{K_{d}}+1\right)\left(\frac{S^{tot}}{K_{S}}+1\right)}\cdot S^{tot}=k_{cat}\cdot \frac{E_{0}^{tot}}{\left(\frac{I_{0}}{K_{d}}+1\right)\left(\frac{S^{tot}}{K_{S}}+1\right)}\cdot S^{tot}$$

Solving Equation (8) with respect to L and F, we arrive at Equation (11) of the main text.

Second, we consider the case when before overexpression of the kinase its abundance was smaller than the substrate abundance, $E_{0}^{tot}\ll S^{tot}$, but after overexpression the kinase abundance becomes greater than the substrate abundance, $LE_{0}^{tot}\gg S^{tot}$. Accordingly, we can neglect the moiety conservation law for the substrate before overexpression, and the moiety conservation law for the kinase after overexpression:

(9) $$E_{0}\left(\frac{I_{0}}{K_{d}}+1\right)\left(\frac{S^{tot}}{K_{S}}+1\right)=E_{0}^{tot}\to \;E_{0}=\frac{E_{0}^{tot}}{\left(\frac{I_{0}}{K_{d}}+1\right)\left(\frac{S^{tot}}{K_{S}}+1\right)}\;S\left(1+\frac{LE_{0}^{tot}}{K_{S}}\left(1+\frac{k_{cat}}{k_{p}}+\frac{FI_{0}}{K_{d}}\right)\right)=S^{tot}\;\to \;S=\frac{S^{tot}}{1+\frac{LE_{0}^{tot}}{K_{S}}\left(1+\frac{k_{cat}}{k_{p}}+\frac{FI_{0}}{K_{d}}\right)}$$

Then, the equality of the phosphorylation rates reads as follows:

(10) $$k_{cat}\cdot LE_{0}^{tot}\cdot \frac{S^{tot}}{1+\frac{LE_{0}^{tot}}{K_{S}}\left(1+\frac{k_{cat}}{k_{p}}+\frac{FI_{0}}{K_{d}}\right)}=k_{cat}\cdot \frac{E_{0}^{tot}}{\left(\frac{I_{0}}{K_{d}}+1\right)\left(\frac{S^{tot}}{K_{S}}+1\right)}\cdot S^{tot}$$

Solving Equation (10) with respect to L and F, we arrive at Equation (12) of the main text.

### 2.9. A Structure-Based Model of the MAPK Pathway

#### 2.9.1. Model Formulation

The RAF family includes three evolutionarily conserved cytosolic serine/threonine kinases (ARAF, BRAF, and CRAF). All paralogs contain three conserved regions: the N-terminal region CR1, comprised of the RAS Binding Domain (RBD) [25] and the cysteine-rich domain (CRD), which stabilize the inactive conformation; region CR2, which contains residues important for RAF membrane recruitment during activation; and region CR3, containing the kinase domain, which includes the catalytic DFG motif, as well as the regulatory αC-helix domain. All members of the RAF family share these conserved regions, but ARAF and CRAF are more similar to each other than to BRAF. Upstream of CR3 is the N-terminal acidic (NtA) region, which, in BRAF (SSDD, residues 446–449), features two negatively charged aspartates and a constitutively phosphorylated Ser446 residue [26,27,28]. The corresponding regions in ARAF (SGYY, 299–302) and CRAF (SSYY, 338–401), however, contain tyrosine residues in place of aspartate, and phosphorylation of Ser and Tyr residues within this region is induced during activation [29]. Phosphorylation of the NtA motif facilitates RAF dimerization through interprotomer salt bridges [30], and is required for allosteric activation of RAF dimers [29]. Accordingly, we have implemented ARAF interactions with BRAF and CRAF in a similar way to CRAF interactions.

Previously developed MAPK pathway models included only the two most well-studied RAF isoforms: BRAF and CRAF [14,17,31]. The model developed in this study was extended by explicitly adding ARAF and formulated using the PySB framework [32], with the support of BioNetGen’s energy-based implementation of ordinary differential equations (ODEs) [33,34,35,36]. All RAF isoforms share the following binding sites and phosphorylation sites: (1) RBD, (2) dimerization domain, (3) inhibitor binding pocket, (4) inhibitory phosphorylation site, (5) site of feedback phosphorylation by ppERK, whereas only CRAF and ARAF have (6) activation phosphorylation sites that enhance the kinase activity as a dimer. ARAF has significantly lower endogenous kinase activity than BRAF or CRAF [37], so we assumed that ARAF kinase activity is negligibly small as a monomer, and it can transmit signaling only when it is in a dimer formation. Overall, the model contains 2216 species and 20,716 reactions. All values of kinetic parameters and thermodynamic factors were derived from our previous studies (cf. “Kds_and_thermodynamic_factors.xlsx” in Supplementary Materials) [17,31].

#### 2.9.2. Allosteric Interactions of RAF Proteins and Inhibitors

Protein kinases, including RAFs, can assume different conformational states of the regulatory structural motifs αC-helix and DFG. The positions of these motifs (termed IN or OUT) correspond to active and inactive kinase conformations, respectively. ATP-competitive RAF inhibitors can be classified based on their preferential binding to different (IN or OUT) conformations of the αC-helix and the DFG motif [38,39,40]. At present, three types of RAF inhibitors, which preferably bind to (1) αC -IN, DFG-IN (CI/DI), (2) αC -IN, DFG-OUT (CI/DO) or (3) αC-OUT, DFG-IN (CO/DI) conformations of RAF molecules, have been produced. Each inhibitor type binds preferably to one out of the possible four αC -IN/OUT, DFG-IN/OUT conformations of RAF molecules. Preferential binding is described by the smallest dissociation constant ($K_{d}$), and each inhibitor can also bind to other conformations [41], yet with much larger $K_{d}$’s. To implement the inhibitor type-specific affinity to RAF molecules and their allosteric effects, we introduced the following thermodynamic factors (Table 1) [31].

#### 2.9.3. Numerical Simulation of the Model

The change in Receptor Tyrosine Kinase (RTK) activities from low to high levels in cells expressing wild-type RAS was modeled identically to our previous work [17]. The equilibrium constant of SOS binding to GRB2 was increased 5-fold (cf. the values of the SOS membrane-cytoplasm distribution parameter KSOStransl in the file “raf_overexp_model.sbml” in Supplementary Materials). To simulate oncogenic RAS mutant conditions, the rate constant of the RAS–GAP activity was decreased 10-fold (cf. the values of the parameter V_RASGAP in the file “raf_overexp_model.sbml” in Supplementary Materials). These parameter changes resulted in stationary RAS–GTP levels equal to ~25 nM in wild-type RAS cells with low RTK activity, and ~250 nM in cells with oncogenic RAS, respectively. The total RAS concentration was set to 750 nM.

The energy-based rules generate corresponding systems of ODEs, which were numerically integrated using ScipyOdeSimulator provided in PySB [32]. We used the ‘vode’ integrator implemented in SciPy [42] with a relative and absolute error tolerance of 10^−8^. For every simulation condition, the model was simulated for 10 h to equilibrate the system. The simulation conditions in each figure, e.g., the initial abundance of RAF isoforms, are provided in their legends.

## 3. Results

### 3.1. Drug Resistance Caused by Overexpression of the Primary Drug Target

We start with a simplified analytical derivation of the drug dose required to inhibit a monomeric kinase that increases its abundance in response to the initial inhibitor exposure. For simplicity, we describe the pharmacological effects of the exposure to inhibitors in vitro, disregarding time-dependent pharmacokinetics effects. Next, using a semi-analytical, simplified model of a kinase that dimerizes, we show that the IC50 (as well as other indicators, such as GI50 or EC50/ED50) demonstrates a steeper upward trend in response to increased enzyme abundance, compared to a kinase that does not dimerize. Then, we use a detailed, structure-based model of the ERK pathway, to demonstrate how resistance to different types of RAF inhibitors depends on (1) a compensatory upregulation of RAF isoforms following RAF inhibition, and (2) knockdown of one RAF isoform and a compensatory upregulation of other isoforms. The model predicts that a combination of two conformation-specific RAF inhibitors can overcome resistance brought about by compensatory RAF upregulation. Finally, we demonstrate that all modelling results can be validated by experimental findings.

#### 3.1.1. Overexpression of Monomeric Kinase

We consider inhibition of a kinase (E) by a drug (I) with the dissociation constant $K_{d}$ (Figure 1A). Given the dose $I_{0}={IC50}_{0}$ that results in 50% of inhibition of the kinase when its abundance is $E_{0}^{tot}$, we calculate the new drug dose (I=IC50) required to maintain the same 50% inhibited reaction rate when the kinase abundance is increased. Notably, the initial drug dose, ${IC50}_{0}$, can be a dose resulting in any fixed percentage of inhibition of the kinase activity, such as ${IC70}_{0}$, etc. When the enzyme concentration is much smaller than the concentration of its protein substrate, the fold-increase in the IC50 drug dose ($I/I_{0}$) linearly depends on the fold-increase in the enzyme abundance ($E^{tot}/E_{0}^{tot}$, see Section 2):

(11) $$\frac{I}{I_{0}}=\frac{\frac{E^{tot}}{E_{0}^{tot}}\cdot \left(1+I_{0}/K_{d}\right)-1}{I_{0}/K_{d}}$$

As the IC50 usually vastly exceeds the dissociation constant, it follows from Equation (11) that the fold-change in the inhibitor dose will practically be equal to the fold-change in enzyme concentration.

A large increase in kinase abundance makes it comparable to or even exceeding the abundance of its protein substrate, violating the standard assumptions of enzyme kinetics. In this case, a nonlinear equation connects the fold-increase in the drug dose, the abundances of the kinase and its substrate, and kinetic parameters (Section 2). However, if overexpression of the kinase makes its abundance substantially greater than the substrate abundance ($S^{tot}$), then the fold-increase of the drug required to keep the same 50% kinase inhibition is approximated as:

(12) $$\frac{I}{I_{0}}=\frac{1-E_{0}^{tot}/E^{tot}}{E_{0}^{tot}I_{0}/\left(K_{S}K_{d}\right)}+\frac{S^{tot}/E_{0}^{tot}-1-k_{cat}/k_{p}}{I_{0}/K_{d}}+\frac{K_{S}+S^{tot}}{E_{0}^{tot}}$$

Here, $K_{S}$ and $k_{cat}$ are the substrate dissociation and catalytic rate constants, and $k_{p}$ is the phosphatase rate (Section 2 Methods, Section 2.7). If the IC50 inhibitor doses greatly exceed the $K_{d}$, this expression simplifies as follows:

(13) $$\frac{I}{I_{0}}=\frac{K_{S}+S^{tot}}{E_{0}}$$

We conclude that the ratio of the total kinase abundance and its protein substrate is a key parameter determining the relation between fold-increase in drug concentration with the fold-increase in kinase abundance (Figure 1B). If this ratio remains small for the overexpressed kinase, the drug dose should increase almost proportionally to the kinase abundance. However, when this ratio becomes very large and, in consequence, the substrate turns out to be a limiting factor, further kinase overexpression does not require additional large increases in drug dose (as illustrated in Figure 1B and Figure S1, for different abundances and kinetic constants).

#### 3.1.2. Overexpression of a Kinase That Dimerizes

We start with a simple, semi-analytical mathematical model of kinase dimerization developed in our previous study [14] (Figure 1C). We consider the following scenarios: (1) the $K_{d}$ value describing dimerization affinity is extremely large, i.e., the concentration of kinase dimers is negligibly small at steady state; (2) no allosteric effects are caused by a drug; and (3) an allosteric inhibitor facilitates kinase dimerization and thus binding of the first inhibitor molecule, but it decreases the affinity for a second drug molecule. The thermodynamic factors f and g describe the inhibitor-induced increase in the dimerization affinity and changes in affinity of the second inhibitor molecule for a dimer, respectively. Kinase dimers, such as RAF dimers, have remarkably higher kinase activity than monomers, even if one (but not both) protomer is inhibitor bound.

Our model shows that, as the kinase abundance increases, the inhibitor concentration required for the same level of inhibition of kinase activity increases more precipitately for kinase dimers, specifically when there are allosteric interactions between kinase and inhibitor, and much lesser for monomeric enzyme (Figure 1D). To summarize, kinase dimerization underlies drug resistance caused by overexpression of the primary drug target, and it can be facilitated by drug-induced allosteric effects. However, to better understand which inhibitor types to use for a pathway inhibition, considering multiple feedback loops and the specific cellular context, we need to extend the model and connect thermodynamic and structural analyses of detailed protein–drug interactions with biochemical, mutational, and pathway regulation data, including the dynamics of posttranslational modifications (PTMs) and feedback loops.

**Figure 2:**
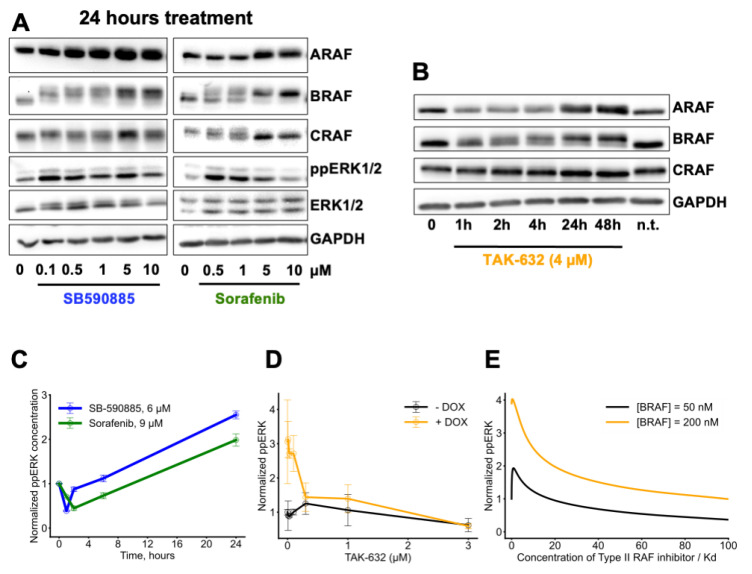
RAF overexpression in *RAS*-mutant cells. (**A**,**B**) RAF inhibitors increase the abundance of RAF isoforms in *NRAS*-mutant cells. (**A**) Growing MEL-JUSO cells were treated with increasing concentrations of Type I RAF inhibitor SB-590885 (**left**) or Type II RAF inhibitor Sorafenib (**right**), as indicated, for 24 h. DMSO served as a control. Cells were lysed and the expression of RAF isoforms and ERK activation were assessed using western blot. One representative replicate is shown. (**B**) Growing MEL-JUSO cells were treated with 4 μM Type II RAF inhibitor TAK-632 for the indicated times and analyzed as in (**A**). n.t.—non treated (**C**,**D**) Overexpression of RAF isoforms contributes to reactivation of ERK signaling. (**C**) Growing MEL-JUSO cells were treated with 6 μM SB-590885 (blue) or 9 μM Sorafenib (green) for the indicated times. Relative ppERK levels were quantified using the MSD Multi-Spot Assay ELISA System. (**D**) Overexpression of BRAF^WT^ in CaCo-2tet cells (RAS^WT^) was induced by addition of doxycycline for 24 h (orange). Uninduced cells (black) were used as control. Subsequently, cells were treated for a further 24 h with increasing amounts of TAK-632. Relative ppERK levels were quantified using Luminex ELISA, as described. The averages of three independent replicates are shown, error bars represent mean ± SEM. (**E**) Dependence of ERK signaling on the RAF abundance for cells with wild-type RAS ([RAS–GTP] = 25 nM). BRAF abundance is set to 50 nM (black) and 200 nM (orange). For both conditions, CRAF and ARAF abundances are set to 50 nM. The model predicts stationary responses of ERK signaling to Type II RAF inhibitor (e.g., TAK-632).

As a proof of concept, we study the RAS/RAF/MEK/ERK pathway. Dimerization of RAF is known to drive resistance to RAF inhibitors. One of suggested resistance mechanisms is the increase in RAS activity, due to the relief of negative feedback in the RAS/RAF/MEK/ERK pathway, following a drug-induced decrease in active ERK. Our data suggest that, regardless of the increase in RAS–GTP levels, which may or may not happen [43], RAF inhibitors increase the abundance of RAF isoforms (Figure 2A,B, and Figure S2A,B), especially at high RAFi concentrations. Both the increase in RAS–GTP levels and RAF abundance result in increased RAF dimer formation. As a result, whereas pathway inhibition is observed at 1 h after drug treatments, pathway reactivation—and often an increase in the original signaling activity—is observed at 24 h (Figure 2C, time series for SB-590885 and Sorafenib). This signaling overshoot cannot be explained by the negative feedback relief [17].

**Figure 3:**
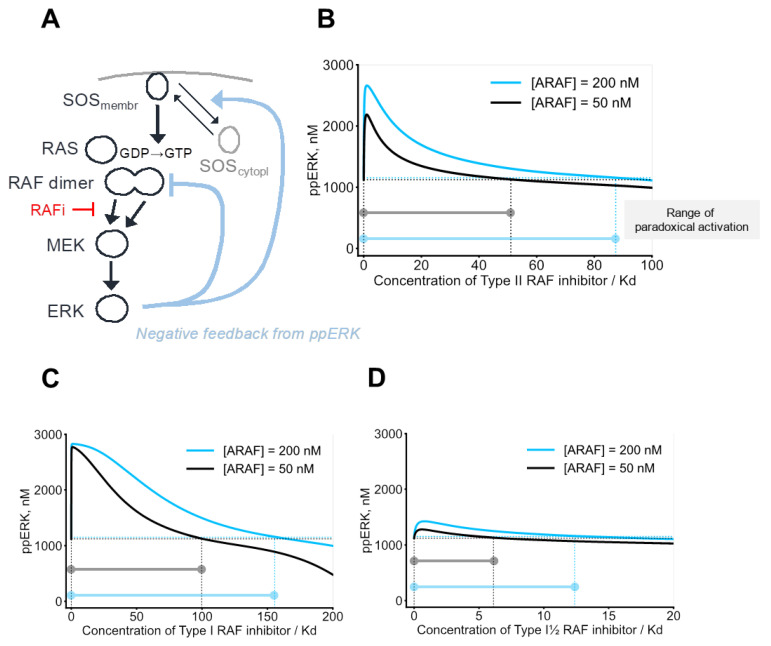
Structure-based model predictions: ARAF isoform upregulation results in the increase in the range of paradoxical activation. (**A**) Schematic overview of the processes described in the model. (**B**–**D**) Dependence of ERK signaling on the ARAF abundance for cells expressing mutant RAS ([RAS–GTP] = 250 nM). ARAF abundance was set to 50 nM (black) and 200 nM (sky blue). For both conditions, BRAF and CRAF abundances were set to 50 nM. Dashed horizontal lines indicate basal ppERK levels for each condition. Vertical dashed lines denote inhibitor concentrations at which dose response curves drop below their basal levels. The model predicts stationary responses of ERK signaling to the Type II RAF inhibitor (**B**), Type I RAF inhibitor (**C**), and Type I½ RAF inhibitor (**D**).

To study the effect of an increase in RAF isoforms, we constructed a structure-based, detailed mechanistic model of the RAS/RAF/MEK/ERK pathway (schematically shown in Figure 3A). The model integrates the existing knowledge for this pathway, including thermodynamics and kinetics of kinase-drug interactions, pathway regulation by feedback loops, PTMs, and protein expression levels. Allosteric effects caused by structurally different RAF inhibitors were incorporated into the model through thermodynamic factors [14], which quantify inhibitor-induced changes in the affinities of kinase dimerization and kinase–drug binding.

RAF inhibitors are classified into three types, based on which conformation of two intramolecular motifs, αC-helix and DFG, they preferably bind: αC-IN/DFG-IN (Type I), αC-IN/DFG-OUT (Type II), or αC-OUT/DFG-IN (Type I½). We calculated ppERK responses to all types of RAF inhibitors at different abundances of RAF isoforms. Our model demonstrated that, in cells with both wildtype and mutant RAS, an increase in ARAF abundance results in an increased range of paradoxical activation for all types of inhibitors (Figure 3B–D). Overexpression of CRAF results in a qualitatively similar effect. Biochemical data demonstrated that αC-IN inhibitors are more effective in blocking RAF dimers compare to αC-OUT inhibitors [39,44]. However, αC-IN inhibitors can promote RAS–RAF interaction, resulting in increased formation of RAF dimers [45]. Our model predictions that Type I and II RAF inhibitors induce a strong paradoxical activation and increase the range of paradoxical activation reflect these mechanisms and are in accordance with our experimental data. Interestingly, overexpression of BRAF, although leading to the increase in ERK activity, decreases the range of paradoxical activation, when compared to the increased basal levels (Figure S3A,B). As BRAF overexpression increases the basal ERK level more powerfully than the ARAF/CRAF overexpression, the relative decrease in the range of paradoxical activation is explained by nonlinear dependence of MEK and ERK activation, showing saturation at very high RAF catalytic activities. In CaCo-2 cells expressing wild-type RAS, doxycycline-induced overexpression of wild-type BRAF results in increased ERK activity at a lower concentration of Type II RAF inhibitor (TAK-632) but decreased the range of paradoxical activation (Figure 2D), which was recapitulated in our model (Figure 2E). In summary, our model was capable of predicting not only inhibitor-specific ERK signaling responses, but also isoform-specific effects on dose responses.

### 3.2. Cells Adapt to the Knockout of One RAF Isoform by Overexpression of Other RAF Isoforms

**Figure 4:**
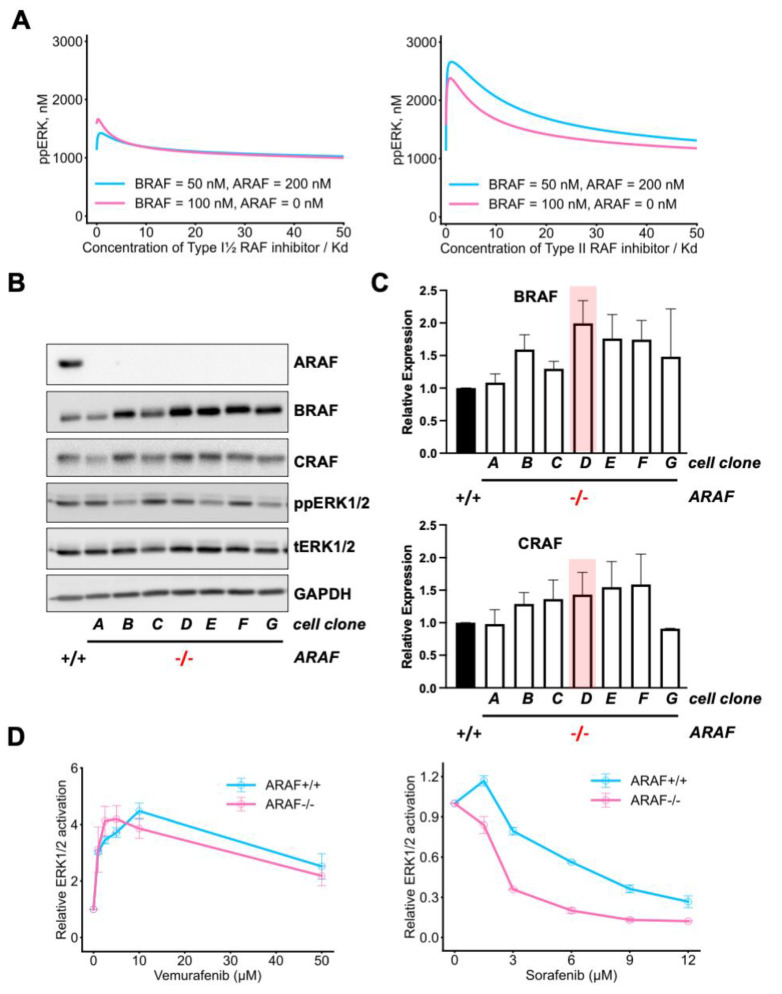
Knockout of one *RAF* isoform results in overexpression of other RAF isoforms to sustain ERK signaling. (**A**) Model predictions of dose responses and their dependencies on the RAF isoform abundances. Effect of Type I½ and Type II RAF inhibitors on ERK phosphorylation under ARAF knockout and BRAF overexpression. (**left**) [BRAF] = [CRAF] = 50 nM, [ARAF]= 200 nM. (**right**) [BRAF] = 100 nM, [CRAF] = 50 nM, [ARAF] = 0 nM. (**B**) *ARAF^+/+^* or single-cell derived CRISPR/Cas9 knockout *ARAF^–/–^* MEL-JUSO cells were lysed, and RAF expression and ERK activation were analyzed by western blot. GAPDH served as a loading control. (**C**) Western blots of BRAF and CRAF expression levels in three replicates were quantified. Bar charts represent relative BRAF or CRAF expression in *ARAF^–/–^* cell clones. Error bars represent mean ± SEM. Clone #D (highlighted in red) was used for the dose response curves shown in (**D**). (**D**) Growing *ARAF^+/+^* or *ARAF^–/–^* MEL-JUSO cells were treated with increasing concentrations of Vemurafenib or Sorafenib as indicated for 24 h. DMSO served as a control. ERK1/2 activation was assessed using the Magpix ELISA system. Charts represent relative ERK1/2 activation, error bars represent mean ± SEM, *n* = 3.

It was shown that *NRAS* mutant melanoma cells can keep their malignant properties after knockout of *BRAF* and *CRAF*, being dependent on ARAF to sustain ERK signaling [46]. Here, we demonstrate that knockout of one of the *RAF* isoforms (*ARAF*) can result in the overexpression of other RAF isoforms to maintain RAF/MEK/ERK signaling (Figure 4 and Figure S4A). This readjustment in RAF abundances results in similar ppERK basal levels being sustained as the parental cells (Figure S4A). Our model confirms that knockout of one of the *RAF* isoforms can be compensated by overexpression of other RAF isoforms, but drug responses may differ depending on the type of inhibitor and its isoform specificity (Figure 4A). These modeling predictions are in line with our experimental results (Figure 4B–D). Importantly, we observed that *ARAF* knockout and the subsequent BRAF overexpression sensitizes cells to Type II RAF inhibitor (Sorafenib), but not to Type I½ RAF inhibitor (Vemurafenib), which cannot inhibit RAF dimers [11,39,47]. In addition, *ARAF* depletion sensitizes cell viability and proliferation to inhibition with Sorafenib, and, more importantly, synergistic Vemurafenib/Sorafenib inhibition (Figure S4B).

Overexpression of one kinase isoform after depletion of the other is a typical example of paralog buffering, widely described in the literature [48]. However, such overexpression happens to a different extent in different clones, and in some clones, BRAF and CRAF are not overexpressed at all, showcasing the stochastic nature of this mechanism.

### 3.3. A Combination of Conformation-Specific RAF Inhibitors Overcomes Adaptation of Oncogenic Cell Signaling

**Figure 5:**
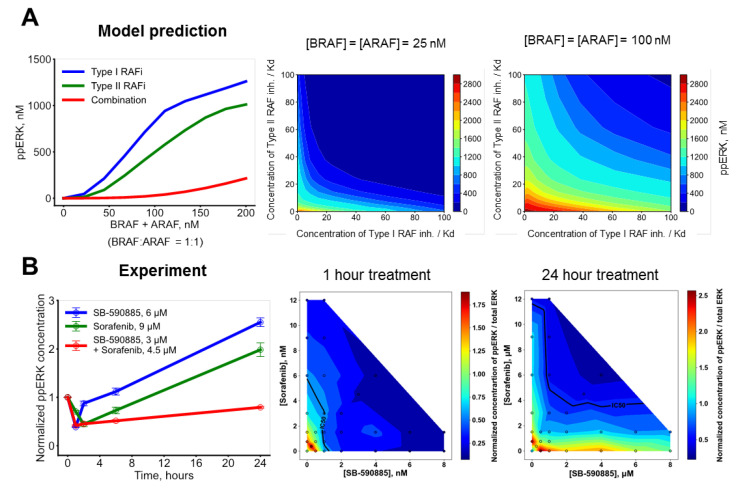
Our model predicts how combination of two RAF inhibitors overcomes drug resistance brought about by RAF overexpression. (**A**) Predictive simulations demonstrate synergy of inhibiting ERK signaling by combinations of Type I and Type II RAF inhibitors in RAF-overexpressing cells. (**Left panel**) Dependencies of ERK activation on BRAF and ARAF abundances for cells treated with 150·Kd Type I RAF inhibitor (blue), 200·Kd Type II RAF inhibitor (green), or a combination of 75·Kd Type I RAF inhibitor and 100·Kd Type II RAF inhibitor (red). The concentration of CRAF is 50 nM. (**Middle and right panels**) Isolines of steady-state ERK signaling responses to type Type I and Type II RAF inhibitors and their combinations. (**Middle panel**) [BRAF] = [ARAF] = 25 nM. (**Right panel**) [BRAF] = [ARAF] = 100 nM. For both conditions, [CRAF] = 50 nM. (**B**) ppERK signaling responses of growing MEL-JUSO cells to two structurally different RAF inhibitors and combination. (**Left panel**) Time course measured using MSD Multi-Spot Assay ELISA System in cells treated with 6 μM SB-590885 (blue), 9 μM Sorafenib (green), or a combination of 3 μM SB-590885 and 4.5 μM Sorafenib. (**Middle and right panels**) Dose responses to SB-590885, Sorafenib, and combination measured using xMAP assay for 1 h (middle) and 24 h of treatment, respectively.

We previously demonstrated that, for drug resistance caused by RAF dimerization, a combination of two RAF inhibitors preferentially binding to different conformations in a RAF molecule is effective [14,31]. As we observed increased concentrations of RAF dimers in response to RAF inhibitors, we hypothesized that a combination of two distinct RAF inhibitors may also be effective to counteract resistance caused by RAF overexpression. To test this, we computed ppERK levels upon treatment with two structurally distinct RAF inhibitors. Experimental data demonstrate that, in response to RAF inhibitors, the abundance of RAF isoforms monotonically increases in both a dose-dependent (Figure 2A) and time-dependent (Figure 2B and Figure S5B) manner. Therefore, we utilized RAF abundance as a parameter for simulation of ppERK responses to different RAF inhibitor types, depending on RAF abundance. Model simulations show that a combination of Type I RAF inhibitor (e.g., SB-590885) and Type II RAF inhibitor (e.g., Sorafenib) can efficiently suppress ERK activity, whereas it cannot be blocked when either RAF inhibitor is used on its own (Figure 5A). To assess the efficiency of ERK inhibition by two drugs, we calculated Loewe isoboles [49] for combinations of a Type I RAF inhibitor and a Type II RAF inhibitor. If two inhibitors work in synergistic manner, then Loewe isoboles are concave, because lower overall concentrations result in the same inhibitory effect, whereas convex isoboles indicate antagonism between inhibitors, because the concentrations must be increased to achieve the same level of inhibition. Our model shows that Loewe isoboles are concave for a wide range of concentrations of these drugs, even when BRAF is overexpressed, indicating synergy between Type I and Type II RAF inhibitors (Figure 5A). Experimental data fully corroborate these modelling predictions, showing that combination of two different types of RAF inhibitors (SB0590885 and Sorafenib) effectively counteracts the overactivation of ERK induced by an increase in the RAF abundance in MEL-JUSO cells (Figure 5B). Due to reactivation, some synergistic effects between RAF inhibitors can be seen very slightly after 1 h but become pronounced at 24 h (Figure 5B). RAF inhibitors inhibiting the RAS-to-ERK signaling pathway on a short timescale of one hour cause pathway reactivation at 24 h (time series for SB and SOR). Our simulations also suggest, and experiments confirm, that a combination of Type I½ (e.g., Vemurafenib) and Type II (e.g., Sorafenib) RAF inhibitors effectively overcomes RAF overexpression-induced resistance (Figure S5), similarly to the combination of Type I and Type II RAF inhibitors.

In summary, our mathematical model and experimental data demonstrate that two conformation-specific RAF inhibitors, though ineffective on their own, when combined, can abolish drug resistance caused by the overexpression of RAF isoforms.

## 4. Discussion

Multiple mechanisms accounting for drug resistance to targeted drugs have been described in recent decades, including secondary mutations that abrogate drug-target binding [50], drug-induced kinase dimerization [6,12,13], and relief of negative feedback mechanisms [51,52]. In this paper, we investigated another, less-studied, mechanism, namely the drug-induced upregulation of drug targets.

We can demonstrate that the overexpression of an enzyme can result in resistance, even if it does not dimerize (Figure 1A,B), but this effect is much more pronounced when an enzyme forms dimers (Figure 1C,D).

Using the RAS-to-ERK pathway as an example, our data indicate that overexpression of RAF isoforms increases the concentration range of paradoxical activation for RAF inhibitors. The model developed here predicts, and experiments confirm, that RAF kinase inhibition by any single RAF inhibitor is overcome by RAF overexpression, but a combination of structurally different RAF inhibitors synergize and efficiently suppress resistance. Earlier studies on the role of RAF dimerization in drug resistance have demonstrated that combinations of inhibitors targeting different conformations of the enzyme are effective [17,31]. In this study, we show that this strategy is also true for resistance caused by increased RAF isoform abundance.

It was shown that ERK signaling is not suppressed after knockout of *ARAF* [53]. Our data indicate that *ARAF* depletion in *NRAS* mutant cells results in overexpression of BRAF to maintain ERK signaling, which is consistent with earlier observations. Moreover, our model prediction and experimental data showed that cells become more sensitive to Type II RAF inhibitor in the absence of ARAF, which is mainly due to the reduction of BRAF:ARAF heterodimers. A recent study by Venkatanarayan et al. demonstrated that *ARAF* ablation in *KRAS* mutant cells (A549 cell line) sensitized cells to Type II RAF inhibitor (AZ-628) [54]. Taken together, our model predictions are not only in line with extensive published experimental data, but also support the hypothesis that levels of CRAF and ARAF impact sensitivity to RAF inhibition.

Our structure-based modeling approach enables us to reveal underlying mechanisms of drug resistance and, importantly, predict response to a combination of different inhibitors. Although we specifically focused on investigating the combinatorial effect of RAF inhibitors, the model assumption is based on fundamental thermodynamic principles and, therefore, the findings derived from this study can be applicable to any kinases that undergo dimerization during physiological activation and facilitated by kinase inhibitors. For example, it has been reported that lapatinib, a small molecule tyrosine kinase inhibitor of both the epidermal growth factor receptor (EGFR) and HER2 [55,56,57], enhances the dimerization of ErbB receptors [58] and induces an upregulation of its target [59]. Based on our model predictions, we thus anticipate that a combination of conformation specific inhibitors can be effective in the context of the ErbB receptor family, as well as in other contexts.

In summary, we present insights into a fundamental drug resistance mechanism caused by the overexpression of the target kinase, and also illuminate the way to overcome it. Incorporating cell-line specific data, as well as the kinetic and thermodynamic properties of protein-protein and protein-drug interactions, into the model, will allow us to predict which drug to use and assess the efficiency of drug combinations for the cell context of interest.

## Figures and Tables

**Table 1:** Description of thermodynamic factors.

Thermodynamic Factor	Description
*f_a_*	The ratio of Kd for binding RAFi1 to the R1 in the R1-R2 dimer versus Kd for binding RAFi1 to the free monomer R.
*f_b_*	The ratio of Kd for binding RAFi2 to the R1 in the R1-R2 dimer versus Kd for binding RAFi2 to the free monomer R.
*g*_1*a*_	The ratio of Kd for RAFi1 binding to the promoter R2 in the R1-R2 dimer versus Kd for the RAFi1 binding to the promoter R1 in the R1-R2 dimer.
*g*_1*b*_	The ratio of Kd for RAFi2 binding to the promoter R2 in the R1-R2 dimer versus Kd for the RAFi2 binding to the promoter R1 in the R1-R2 dimer.
*g*_2*a*_	The ratio of Kd for RAFi1 binding to the protomer R1 in the R1-R2-RAFi1 dimer versus Kd for the RAFi1 binding to the free monomer R.
*g*_2*b*_	The ratio of Kd for RAFi2 binding to the protomer R1 in the R1-R2-RAFi2 dimer versus Kd for the RAFi2 binding to the free monomer R.
*g*_3*a*_	The ratio of Kd for RAFi1 binding to the protomer R1 in the R1-R2-RAFi2 dimer versus Kd for the RAFi1 binding to the free monomer R.
*g*_3*b*_	The ratio of Kd for RAFi2 binding to the protomer R1 in the R1-R2-RAFi1 dimer versus Kd for the RAFi2 binding to the free monomer R.

## Data Availability

The model SBML file (‘raf_overexp_model.sbml’) is available in Supplementary Materials. The inhibitor-specific parameters and thermodynamic factors can be found in ‘Kds_and_thermodynamic_factors.xlsx’ file in Supplementary Materials.

## Supplementary Materials

The following supporting information can be downloaded at: https://www.mdpi.com/article/10.3390/biom13081212/s1, Figure S1: Fold-changes in the drug dose (e.g., IC50) required to maintain the same inhibition level (e.g., 50%) are presented versus fold-changes in the abundance of the primary target for different values of key parameters; Figure S2: RAF inhibitors increase the abundance of RAF isoforms in *NRAS*-mutant cells; Figure S3: Model predicts dependence of ERK signaling on the BRAF abundance for cells with mutant RAS; Figure S4: *ARAF* depletion sensitizes cell viability to inhibition with Sorafenib and Vemurafenib/Sorafenib; Figure S5: A combination of Type I½ and Type II RAF inhibitors effectively overcomes drug resistance brought about by RAF overexpression.
