# Supplementary material for: A Combination of Conformation-Specific RAF Inhibitors Overcome Drug Resistance Brought about by RAF Overexpression
Source: Biomolecules. 2023 Aug 2;13(8):1212. doi: 10.3390/biom13081212 (PMC10452107; doi:10.3390/biom13081212)
Supplement: Supplementary file 1 [file biomolecules-13-01212-s001.zip › biomolecules-2437668-supplementary.pdf]

Supplemental Figures

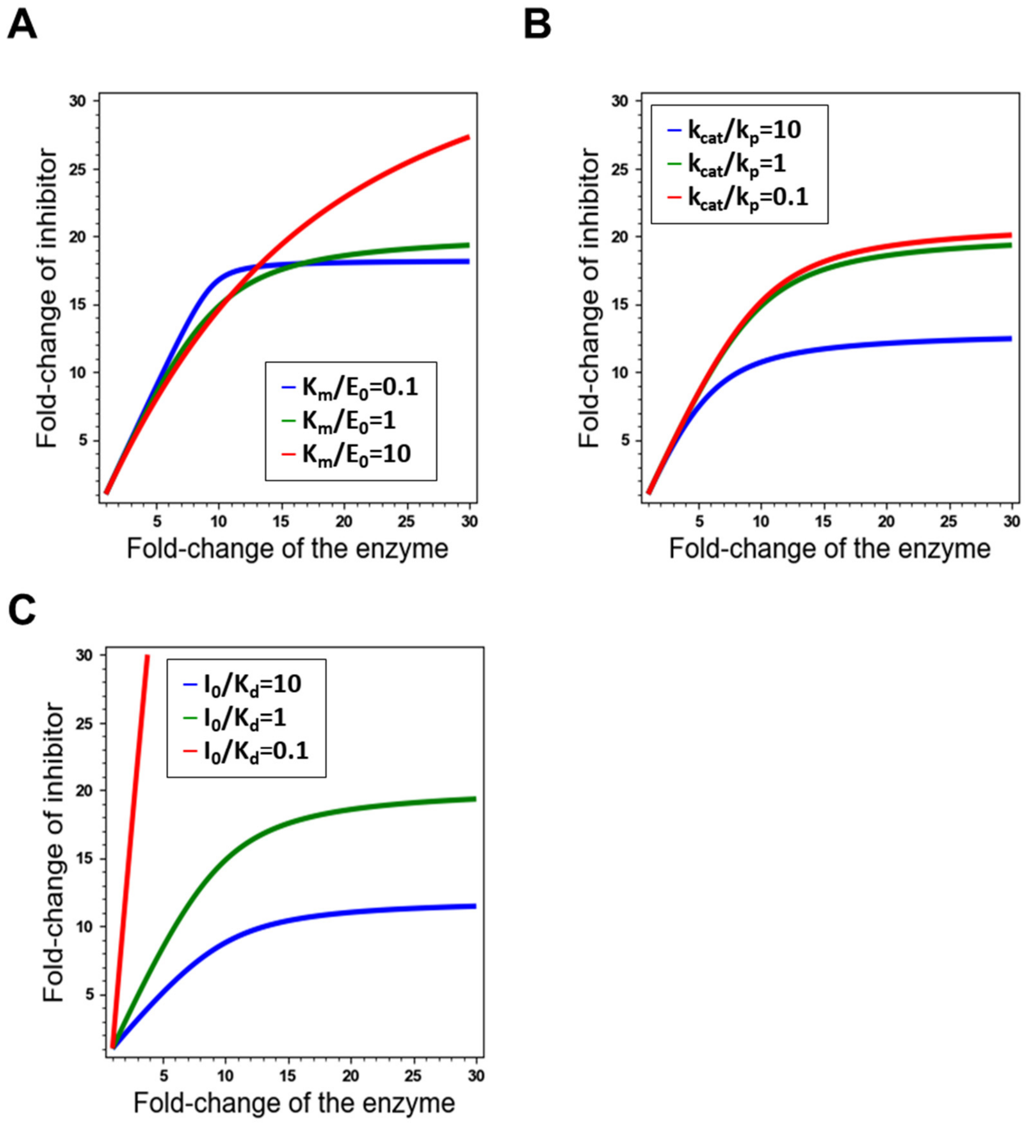

Supplementary Figure S1, Related to Figure 1

(**A-C**) Fold-changes in the drug dose (e.g., IC50) required to maintain the same inhibition level (e.g., 50%) are presented versus fold-changes in the abundance of the primary target for different values of key parameters (see section 2, Materials and Methods).

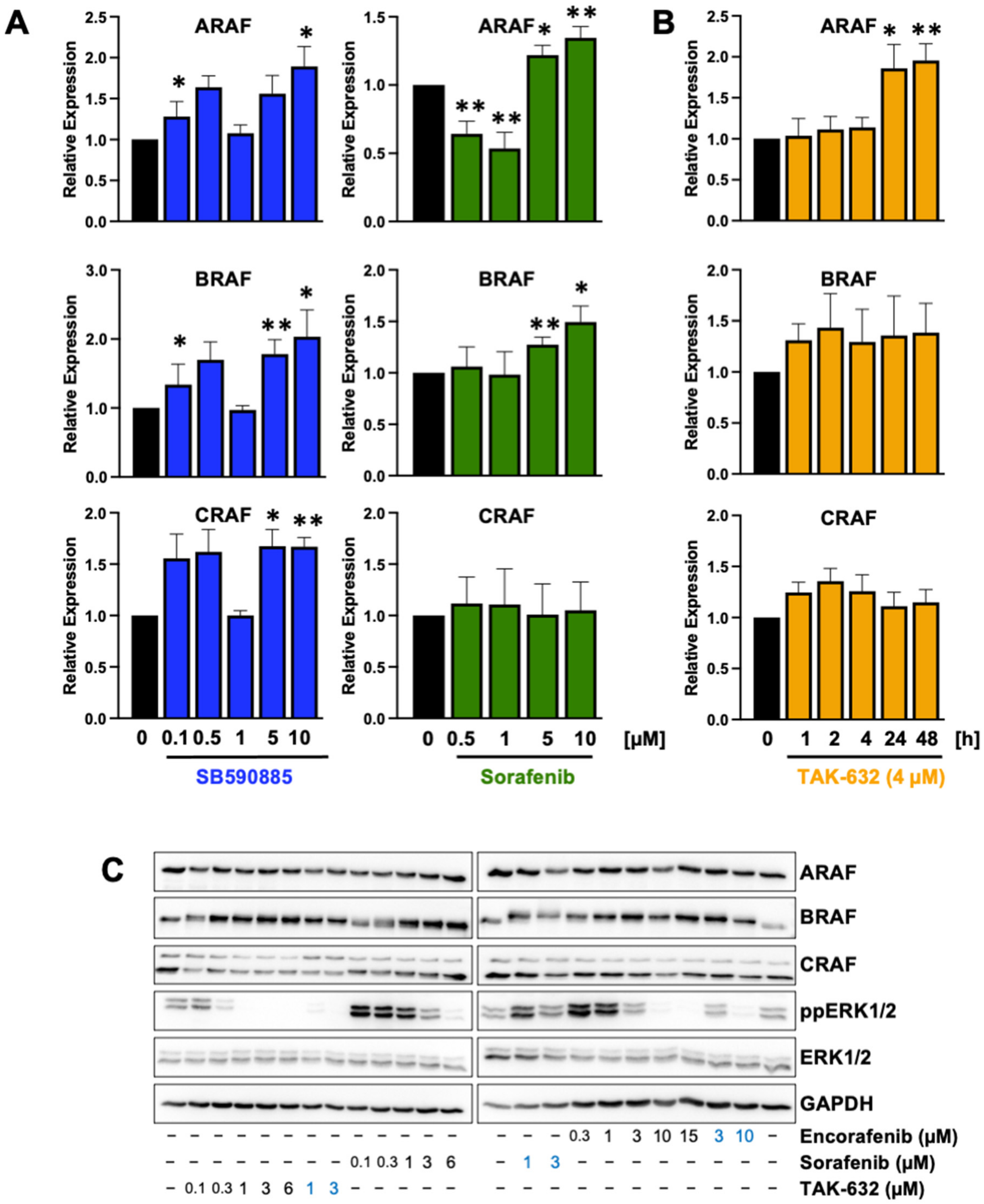

Supplementary Figure S2, Related to Figure 2

(A,B) Quantification of relative ARAF, BRAF, and CRAF expression by Western blot shown in Fig. 2A,B. Bars indicate mean expression with S.E.M. Sorafenib and TAK-632 n=4; SB590885 n=5. Student's T-test. * $p < 0.05$; ** $p < 0.01$. (C) Growing OCI-AML-3 cells were treated with Encorafenib, Sorafenib, and TAK-632, either individually or in combination, for 24 hours (black) or 48 hours (blue). DMSO served as a control. Cells were lysed and expression of RAF isoforms as well as ERK activation were assessed using Western Blotting. One representative replicate is shown.

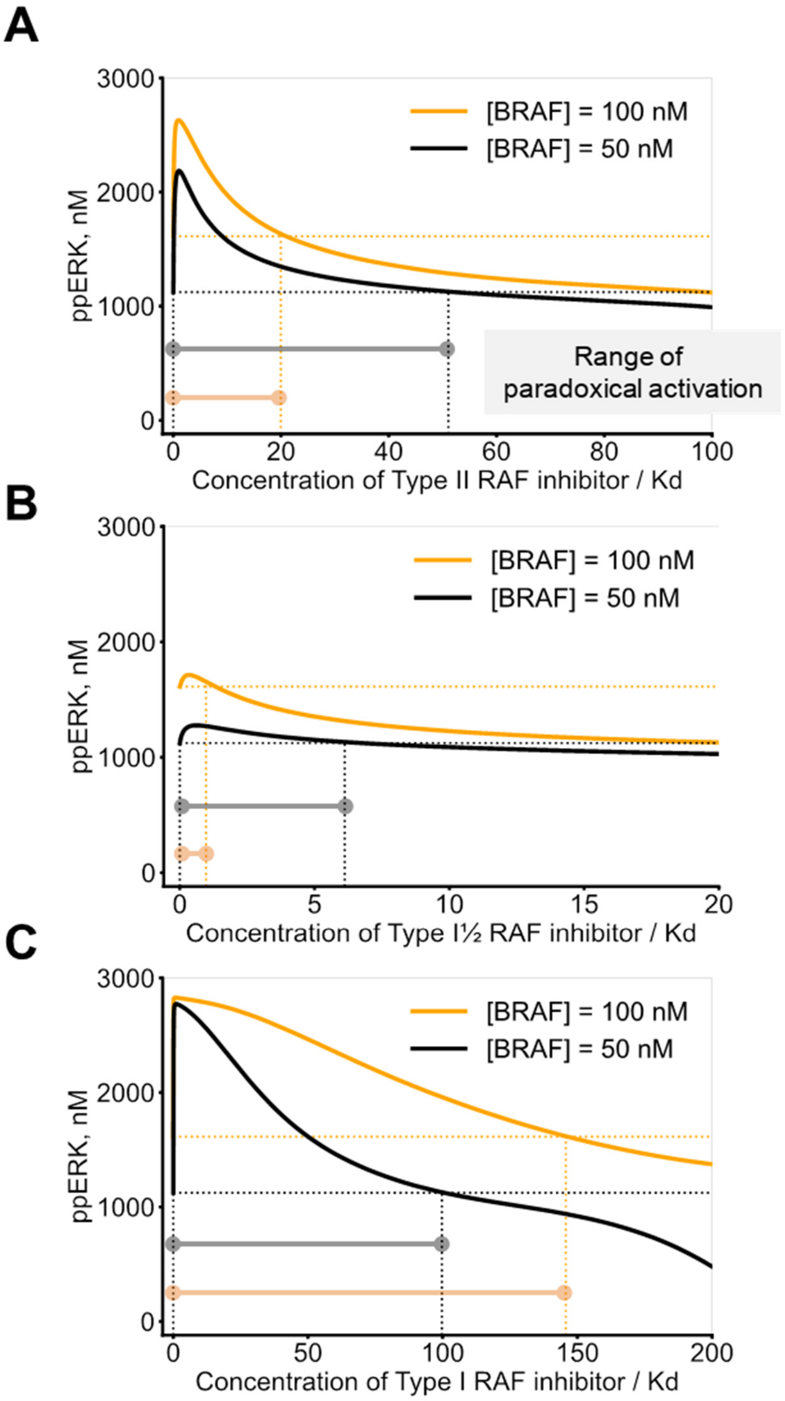

Supplementary Figure S3, Related to Figure 3

(**A-C**) Dependence of ERK signaling on the BRAF abundance for cells with mutant RAS ([RAS-GTP]=250 nM). BRAF abundance is set to 50 nM (black) and 100 nM (orange). For both conditions, CRAF and ARAF abundances are set to 50 nM. Model predicts stationary responses of ERK signaling to Type II RAF inhibitor (**A**), Type I½ RAF inhibitor (**B**), and Type I RAF inhibitor (**C**). Dashed horizontal lines indicate basal ppERK levels for each condition. Vertical dashed lines denote inhibitor concentrations at which dose-response curves drop below their basal levels.

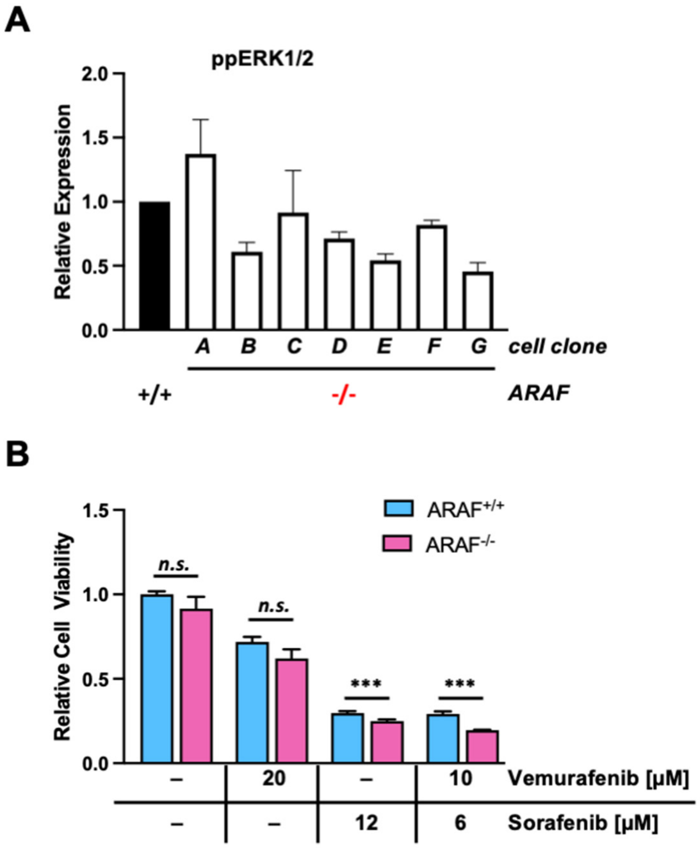

Supplementary Figure S4, Related to Figure 4

(**A**) ARAF+/+ or single-cell derived CRISPR/Cas9 knockout ARAF-/- MEL-JUSO cells were lysed, and RAF expression and ERK activation analyzed by immunoblotting. GAPDH served as a loading control. ppERK1/2 and total ERK1/2 expression of replicates (n=3) was quantified. Bar charts represent relative ERK1/2 activation in ARAF-/- cell clones. Error bars represent mean ±SEM. **(B)** ARAF+/+ or single-cell derived CRISPR/Cas9 knockout ARAF-/- MEL-JUSO cells (clone #D) (n=3) were treated with Vemurafenib, Sorafenib or combinations as indicated, and cell viability assessed by MTS assay 72h post treatment. DMSO served as control. Bar charts represent relative cell viability. Error bars represent mean ±SD. Student's T-test. *** $p < 0.001$.

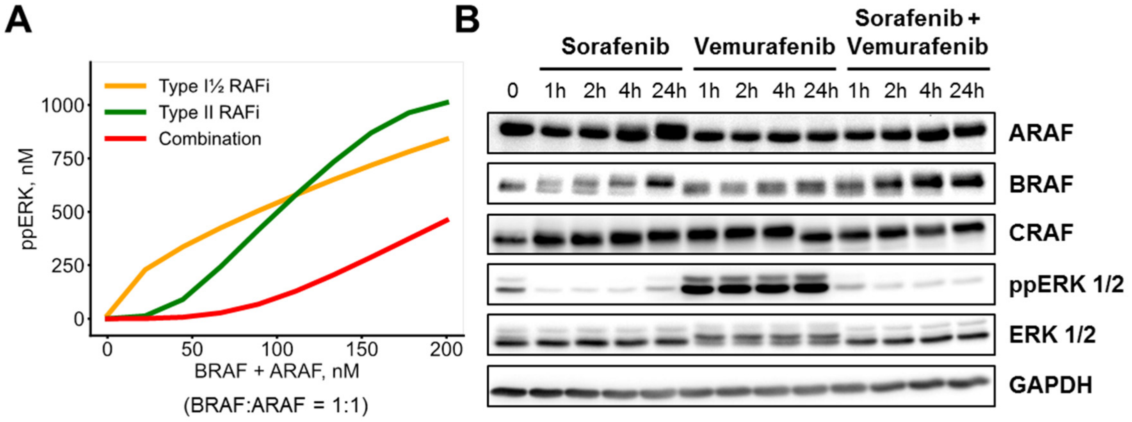

Supplementary Figure S5, Related to Figure 5

(**A**) Model-predicted response of ppERK levels on BRAF and ARAF abundances for mutant RAS ([RAS-GTP]=250 nM) cells treated with 200·Kd Type I½ RAF inhibitor (e.g., Vemurafenib, orange curve), 200·Kd Type II RAF inhibitor (e.g., Sorafenib, green curve), and combination of 100·Kd Type I½ RAF inhibitor and 100·Kd Type II RAF inhibitor (red curve). The concentrations of CRAF and ARAF are both set to 50 nM. (**B**) Growing MEL-JUSO cells were treated with 20 μM Vemurafenib, 9 μM Sorafenib, or a combination of 10 μM Vemurafenib and 4.5 μM Sorafenib for 24 hrs. DMSO served as a control. Cells were lysed and expression of RAF isoforms as well as ERK activation were assessed using Western Blot. One representative replicate is shown.
